# Structural brain abnormalities in children and adolescents with comorbid autism spectrum disorder and attention-deficit/hyperactivity disorder

**DOI:** 10.1038/s41398-019-0679-z

**Published:** 2019-12-09

**Authors:** Yoshifumi Mizuno, Kuriko Kagitani-Shimono, Minyoung Jung, Kai Makita, Shinichiro Takiguchi, Takashi X. Fujisawa, Masaya Tachibana, Mariko Nakanishi, Ikuko Mohri, Masako Taniike, Akemi Tomoda

**Affiliations:** 1 Department of Child and Adolescent Psychological Medicine, University of Fukui Hospital, Fukui, Japan; 2 Department of Psychiatry & Behavioral Sciences, Stanford University School of Medicine, Stanford, USA; 3 Molecular Research Centre for Children’s Mental Development, United Graduate School of Child Development, Osaka University, Suita, Japan; 4 Division of Developmental Neuroscience, United Graduate School of Child Development, Osaka University, Suita, Japan; 5 Department of Paediatrics, Osaka University Graduate School of Medicine, Suita, Japan; 6 Division of Developmental Higher Brain Functions, United Graduate School of Child Development, University of Fukui, Fukui, Japan; 7 Department of Neuropsychiatry, Faculty of Medical Sciences, University of Fukui, Fukui, Japan; 8 Research Centre for Child Mental Development, University of Fukui, Fukui, Japan

**Keywords:** ADHD, Neuroscience, Autism spectrum disorders

## Abstract

Autism spectrum disorder (ASD) and attention-deficit/hyperactivity disorder (ADHD) share high rates of comorbidity, with the Diagnostic and Statistical Manual of Mental Disorders-Fifth Edition now acknowledging the comorbid diagnosis of ASD and ADHD. Although structural abnormalities in the prefrontal cortex, cerebellum, and basal ganglia occur in both ASD and ADHD, no structural studies have focused exclusively on patients with comorbid ASD and ADHD. We thus aimed to clarify the structural features and developmental changes in patients with comorbid ASD and ADHD in a relatively large sample from two sites. Ninety-two patients were age-matched to 141 typically developing (TD) controls (age range: 5–16 years) and assessed for volumetric characteristics using structural magnetic resonance imaging (i.e. surface-based morphometry). While there were no significant differences in prefrontal cortex, cerebellum, and basal ganglia volumes, patients with ASD and ADHD exhibited significantly lower left postcentral gyrus volumes than TD controls. We observed significantly lower postcentral gyrus volumes exclusively in children and preadolescents, and not in adolescents. Our findings suggest that abnormal somatosensory, attributed to delayed maturation of the left postcentral gyrus, leads to the core symptoms experienced by patients with comorbid ASD and ADHD.

## Introduction

Autism spectrum disorder (ASD) is a neurodevelopmental disorder characterized by social impairments, communication deficits, restricted interests, and repetitive behaviours^1^. Attention-deficit/hyperactivity disorder (ADHD), another neurodevelopmental disorder, is likewise characterized by age-inappropriate inattention, hyperactivity, and impulsivity^1^. The prevalence of both disorders is high, with recent reports indicating that ASD is found in 1.5%^2^ of the population, while ADHD is found in 7.2%^3^. ASD and ADHD are more common in boys than in girls, with a male-to-female ratio of about 3–4:1^4,5^. Individuals with ASD or ADHD have difficulties in daily life and often develop a variety of comorbidities such as oppositional defiant disorder, conduct disorder, depression, and anxiety disorder, especially during adolescence or later^1^. It is therefore critical to diagnose and treat these patients early, to prevent the development of secondary psychiatric problems.

The diagnostic criteria outlined in the Diagnostic and Statistical Manual of Mental Disorders-IV (DSM-IV) did not allow for a simultaneous diagnosis of ASD and ADHD in the same individual^6^. Previous studies performed in accordance with the DSM-IV, therefore, did not recognize the occurrence of comorbid ADHD and ASD, leading to ADHD traits in patients with ASD being largely ignored and such patients diagnosed with ASD only. However, clinical symptoms of inattention and hyperactivity often occur in individuals with ASD, and children and adolescents with ADHD often exhibit social interaction deficits^7^, with 28% of patients diagnosed with ASD reported to likely also have comorbid ADHD^8^ and up to 70% of patients with ADHD exhibiting ASD symptoms^9^.

Multiple studies have also suggested that ASD and ADHD are in fact distinct conditions^10^. For example, one study reported that children with ADHD and ASD exhibited greater attention deficits than children with ASD alone^11^. Another study reported that children with ASD and ADHD had greater impairments in adaptive functioning and a poorer health-related quality of life than children with ASD or ADHD alone^12^. In both ASD and ADHD, effective use of stimulant medications can reduce ADHD symptoms, including hyperactivity and inattentiveness, while the behavioural symptoms of ASD remain unchanged^13^. These findings provide further evidence that ADHD and ASD are two distinct disorders. As a result of this distinction between ASD and ADHD, their comorbid diagnosis is now allowed by the DSM-5^1^. Despite high comorbidity and greater impairment experienced by individuals with both conditions rather than a single diagnosis, brain imaging studies of individuals with co-occurring ADHD and ASD are limited and the underlying pathophysiology remains unclear.

Prior brain imaging studies of individuals with ASD or ADHD have revealed some of the pathophysiology as well as biomarkers for these diagnoses. In particular, structural magnetic resonance imaging (MRI) studies have revealed widespread changes in the volume of the cerebral cortex in ASD or ADHD, although these findings are somewhat inconsistent^14^. While ASD is often associated with brain overgrowth in early childhood and adolescence^15^, individuals with ADHD often exhibit smaller brain volumes^16^. The prefrontal cortex and basal ganglia (e.g. the caudate) exhibit increased volumes in ASD^17–19^, and the same regions exhibit decreased volumes in individuals with ADHD^14,20,21^. In contrast, the cerebellum and the corpus callosum exhibit decreased volumes in both ASD and ADHD^14,22–24^. Taken together, these studies indicate that the cerebellum and corpus callosum are affected in similar ways in ASD and ADHD, while findings on total brain, prefrontal cortex, and basal ganglia volumes have shown opposite patterns in the two diseases. How brain volumes are affected in patients with comorbid ASD and ADHD remains unclear.

Although many structural studies of individuals with ASD or ADHD have been reported, as described above, none have so far focused on patients with both ASD and ADHD. Due to this paucity of research, the structural and developmental features of patients with this comorbidity are still a mystery. The purpose of the present study was thus to clarify the structural features and developmental changes that occur in patients with both ASD and ADHD by examining a relatively large structural MRI sample collected across two study sites.

## Materials/subjects and methods

### Participants

The protocol for the present study was approved by the Ethics Committee of the University of Fukui (Assurance no. 20110104) and the Institutional Review Board of Osaka University Hospital (No. 12168-9). All experimental procedures were conducted in accordance with the Declaration of Helsinki and the Ethical Guidelines for Clinical Studies of the Ministry of Health, Labour, and Welfare of Japan. After a complete explanation of the study, all participants and their parent(s) provided written informed consent and assent for participation. This study has been registered with the University Hospital Medical Information Network (UMIN000025830).

In total, this study included 233 participants, 92 boys with ASD and ADHD (ASD + ADHD) and 141 typically developing (TD) boys. Participants with ASD + ADHD were recruited at the University of Fukui or at Osaka University, Japan. TD children were recruited from the local community and assessed to ensure that none had developmental delays, received any special support education, or had a history of epilepsy or other psychiatric disorders. The diagnoses of ASD and ADHD were based on the DSM-5^1^. Participants’ intellect was assessed via the Wechsler Intelligence Scale for Children-Forth or -Third Edition (WISC-IV or WISC-III)^25^. Parents were asked to complete the ADHD Rating Scale-IV (ADHD-RS) to evaluate their child’s inattention and hyperactivity/impulsivity symptoms^26^. The Autism-Spectrum Quotient (AQ) Children’s Version was used at the University of Fukui^27^, and the Social Communication Questionnaire (SCQ) was used at Osaka University to evaluate ASD traits^28^. Handedness was assessed using the Edinburgh Handedness Inventory^29^. Exclusion criteria for both groups included contraindications for MRI, a Full-scale Intelligence Quotient (FSIQ) score < 70, a history of severe head trauma, major physical or neurological illnesses, and drug or substance abuse. The non-autistic and non-ADHD status of TD participants was confirmed using AQ or SCQ and ADHD-RS, respectively.

### Imaging data acquisition

MRI was performed at both the University of Fukui and Osaka University. At the University of Fukui, a 3-T MR scanner (Discovery MR 750; General Electric Medical Systems) was used for high-resolution T1-weighted anatomical MRI (repetition time = 6.38 ms, echo time = 1.99 ms, flip angle = 11°, field of view = 256 × 256 mm^2^, 256 × 256 matrix size, 172 slices, voxel dimensions = 1.0 × 1.0 × 1.0 mm^3^). At Osaka University, two kinds of 3-T MR scanners (Discovery MR 750w and Signa Excite HDxt; General Electric Medical Systems) were used for high-resolution T1-weighted anatomical MRI (repetition time = 880 ms, echo time = 0.016 ms, flip angle = 5°, field of view = 256 × 256 mm^2^, 256 × 256 matrix size, 480 slices, voxel dimensions = 0.94 × 0.94 × 0.94 mm^3^; and repetition time = 10.084 ms, echo time = 3.04 ms, flip angle = 18°, field of view = 512 × 512 mm^2^, 512 × 512 matrix size, 248 slices, voxel dimensions = 0.43 × 0.43 × 0.43 mm^3^).

### Data pre-processing

A vertex model was used to measure the participants’ brain volumes across several thousand cortical folding patterns within a brain surface curvature template^30^, and the FreeSurfer version 6.0 software package (available from: http://surfer.nmr.mgh.harvard.edu/) was used. As in our previous work^31^, pre-processing of anatomical data was performed using the following steps: (1) motion correction and non-uniformity correction, (2) automatic Talairach transformation, (3) intensity normalization, (4) skull strip and segmentation of the subcortical white and grey matter, (5) tessellation of the white and grey matter, (6) surface smoothing and inflation, (7) topology correction, and (8) parcellation. Cortical reconstructions were reviewed for quality and corrected by trained experts, as necessary. Volumes were calculated across 68 regions using the Desikan–Killiany atlas template^32^. Motion artefacts and automated segmentation results were reviewed for data quality by two trained experts (Y.M. and M.J.)^33^. Cortical thickness and surface areas were also investigated, using similar methods.

### Statistical analyses

Demographic data are expressed as means ± standard deviation (SD). All clinical values were compared between groups using Welch’s *t*-test for numerical variables and chi-square tests for categorical variables. All statistical tests were two-tailed; *p-*values < 0.05 were considered statistically significant.

For brain imaging analyses, total intracranial, grey matter, cortical, subcortical grey matter, and cerebellar cortex volumes were compared via analyses of covariances (ANCOVAs) (ASD + ADHD versus TD) that included age, FSIQ, and scan site (i.e. MRI machine) as covariates in the model to control for potential confounding effects. Next, we compared the basal ganglia (nucleus accumbens, amygdala, caudate, hippocampus, pallium, putamen, and thalamus) and calculated cortical volumes, cortical thickness, and surface areas across the 68 regions of interest (ROIs) using the Desikan–Killiany atlas template and ANCOVAs with age, FSIQ, scan site (MRI machine), and total intracranial volume as covariates. The statistical threshold was set to *p* < 0.05 with false discovery rate (FDR) correction for multiple comparisons.

We then separated participants into three groups according to their age, as has been done previously:^34^ children of 5–9 years of age (TD, *n* = 48; ASD + ADHD, *n* = 27), preadolescents of 10–12 years of age (TD, *n* = 59; ASD + ADHD, *n* = 36), and adolescents of 13–18 years of age (TD, *n* = 34; ASD + ADHD, *n* = 29). We compared the volumes of ASD + ADHD-related regions which showed significant differences in the primary analysis, within each age group, between the participants with ASD + ADHD and TD controls using ANCOVAs with FSIQ, scan site (MRI machine), and total intracranial volume as covariates, to investigate the developmental changes. All statistical analyses were performed using the Statistical Package for the Social Sciences 25 software (SPSS, Chicago, IL).

## Results and discussion

### Demographic and clinical characteristics

Participants’ demographic and clinical characteristics are presented in Table 1. In total, 233 subjects including 92 boys with ASD + ADHD (mean age, 11.4 years; SD, 2.4 years) and 141 TD controls (mean age, 11.1 years; SD, 2.5 years) participated in this study. Most participants were right-handed, except for 10 patients with ASD + ADHD and seven TD controls. The ASD + ADHD group included 17 patients with specific learning disorders, 13 with oppositional defiant disorder, three with conduct disorder, two with epilepsy, one with developmental coordination disorder, and one with tic disorder. Forty of the patients with ASD + ADHD were medication-naïve, while 37 were medicated with osmotic release oral system-methylphenidate, ten with atomoxetine, nine with antipsychotics, four with carbamazepine, and one with guanfacine. Although there were no significant differences in age or handedness between the two groups, there were significant differences in IQ, inattention, hyperactivity-impulsivity scores as evaluated by the ADHD-RS and AQ, as well as in SCQ total scores (all *p* < 0.01; Table 1).

**Table 1:** Demographic data of the participants.

	TD	ASD + ADHD	*p*
Subjects (*n*)	141	92	–
University of Fukui (*n*)	69	44	–
Osaka University1 (*n*)	50	31	–
Osaka University2 (*n*)	22	17	–
Age (years)	11.1 (2.5)	11.4 (2.4)	0.47
Sex (*n*, male/female)	141/0	92/0	–
Handedness (*n*, R/L/B)	135/5/2	85/8/2	0.22
FSIQ	109.7 (13.0)	100.5 (15.3)	<0.01
ADHD-RS (total)	3.8 (4.2)	28.0 (9.9)	<0.01
ADHD-RS (IN)	2.8 (3.1)	17.4 (5.2)	<0.01
ADHD-RS (HI)	1.0 (1.5)	10.6 (6.2)	<0.01
AQ-total	11.2 (4.6)	25.8 (6.8)	<0.01
SCQ	4.8 (1.8)	14.6 (6.4)	<0.01

*TD* typically developing, *ASD* autism spectrum disorder, *ADHD* attention-deficit/hyperactivity disorder, *R* right, *L* left, *B* both, *FSIQ* Full-Scale Intelligence Quotient, *ADHD-RS* ADHD-rating scale, *IN* inattention, *HI* hyperactivity/impulsivity, *AQ* Autism-Spectrum Quotient, *SCQ* Social Communication Questionnaire.

### Structural features

**Fig. 1:**
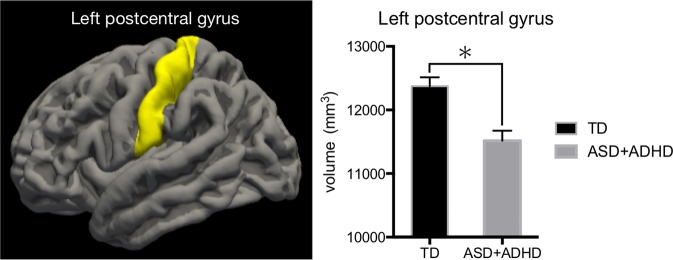
Regions showing significant differences in volume between the ASD + ADHD and TD groups. The ASD + ADHD group had significantly lower left postcentral gyrus volumes than the TD control group. TD typically developing, ASD autism spectrum disorder, ADHD attention-deficit/hyperactivity disorder; *p < 0.05.

**Fig. 2:**
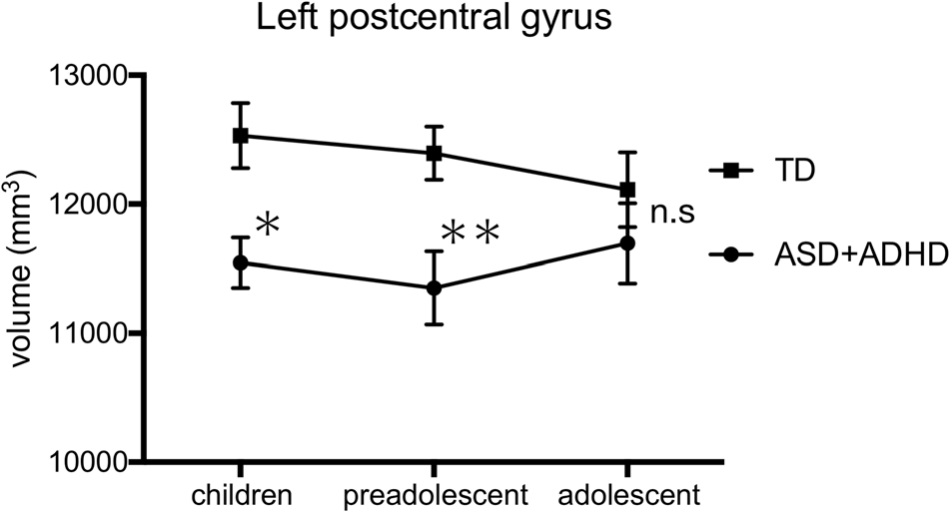
The volume of the left postcentral gyrus in the three developmental groups (children, preadolescents, and adolescents). TD typically developing, ASD autism spectrum disorder, ADHD attention-deficit/hyperactivity disorder; *p < 0.05; **p < 0.01; n.s., not significant.

There were no significant differences in total intracranial, total grey matter, total cortical, subcortical grey matter, or cerebellum cortex volumes between the two groups (Table 1S). Similarly, there were no significant differences in the volumes of the basal ganglia, such as the nucleus accumbens, amygdala, caudate, hippocampus, pallium, putamen, and thalamus (Table 2S). However, compared to TD controls, patients with ASD + ADHD exhibited significantly decreased volumes in the left postcentral gyrus (*p* = 0.018, FDR-corrected) (Fig. 1). There were no significant differences in cortical thickness and surface area between regions. The volumes, cortical thickness, and surface areas of all regions are listed in the supplementary materials (Tables 3S, 4S, 5S).While the left postcentral gyrus volume was significantly lower in children and preadolescents with ASD + ADHD than in TD controls (*p* = 0.026, 0.004), there was no significant difference between patients and controls in the adolescent age group (*p* = 0.171) (Fig. 2).

In the present study, we investigated the structural features of the brain in children with ASD + ADHD using relatively large participant pools from two study sites. While we found no significant differences in prefrontal cortex, cerebellum, or basal ganglia volumes, patients with ASD + ADHD exhibited significantly smaller left postcentral gyrus than TD controls. Additionally, in patients with ASD + ADHD, the volume of the left postcentral gyrus was only significantly smaller in children and preadolescents, not in adolescents.

Few postcentral gyrus volume abnormalities have been previously reported in patients with ASD or ADHD. One study that included only 15 children and adolescents with ADHD and 15 aged-matched participants with ASD revealed increased postcentral gyrus volumes in these patients relative to the TD controls. However, this finding did not survive a correction for multiple comparisons^35^. On the other hand, another study reported that 29 children and adolescents with ADHD exhibited reduced volume in the postcentral gyrus compared to 29 TD controls^36^. This finding survived familywise error corrections. Thus, although prior reports point to structural abnormalities in the postcentral gyrus in ASD and ADHD, findings have been inconsistent. This may be due to the small sample sizes and differences in statistical thresholds used. In the present study, however, we used a relatively large sample size across two recruitment sites, and we report findings after multiple corrections. Our study may thus be able to add more valid evidence to the earlier reported inconsistent findings. In addition, the postcentral gyrus, which is the location of the primary somatosensory cortex, is involved in somatosensory functions. Sensory problems are core symptoms of ASD and are listed in the ASD diagnostic criteria of the DSM-5^1^. Interestingly, children with comorbid ASD and ADHD reportedly experience more sensory processing problems than those with either ASD or ADHD alone^37^, and postcentral gyrus abnormalities may further reflect this increased risk for sensory processing deficits. Furthermore, sensory processing abnormalities contribute to social, communication, and repetitive behaviour deficits, which are the core features of ASD^38^ and are also associated with attentional deficits^39^. Therefore, in children with comorbid ASD and ADHD, sensory problems may stem from postcentral gyrus abnormalities, which in turn may lead to core ASD and ADHD symptoms (social, communication, repetitive behaviour, and attentional deficits).

Critically, developmental trajectories may also be different in patients with ASD and ADHD comorbidity. In the present study, the left postcentral gyrus volume was significantly lower in children and preadolescents with ASD + ADHD than in TD, but there was no difference in adolescents. This finding suggests that developmental delays in the left postcentral gyrus may improve with age. Although ASD and ADHD are lifelong disorders, their features can change with development, and a substantial proportion of individuals with ADHD and a smaller proportion of individuals with ASD exhibit improved symptoms with age^40–42^. These changes in symptoms may result from improvements in postcentral gyrus function with development. Additionally, longitudinal studies of brain development in ADHD have reported delayed cortical development^43,44^. Similarly, as suggested by the present study, brain structure maturation may also be delayed in patients with ASD + ADHD.

Interestingly, we found no significant differences in total brain, cerebellar, prefrontal cortex, corpus callosum, or basal ganglia volumes, although these are areas in which abnormalities are often reported in both ASD and ADHD^14,45,46^. This suggests that the primary pathophysiology in ADHD + ASD may differ from that in ASD or ADHD alone. For instance, in an fMRI study using a temporal discounting task, which assesses a key aspect of reward-related decision making, patients with comorbid ASD and ADHD exhibited more pronounced differences in brain-behaviour associations compared to TD controls and those with ASD or ADHD alone. This finding suggests that ASD and ADHD are neither phenocopies nor additive pathologies, but that comorbid ASD and ADHD represents a distinct neurofunctional pathology altogether^47^. Furthermore, response rates to methylphenidate in individuals with ASD + ADHD differ from those of children with ADHD alone. The National Institute of Mental Health Collaborative Multisite Multimodal Treatment Study of Children with ADHD reported response rates of 70–80%, as compared to the 49% reported in the Research Units of Paediatric Psychopharmacology Autism Network methylphenidate trial^48,49^. Methylphenidate acts to increase dopamine and noradrenaline concentrations by inhibiting their reuptake in the prefrontal cortex and reward system, both of which exhibit abnormalities in patients with ADHD. The less pronounced effect of methylphenidate in patients with ASD + ADHD may thus be due to the attention-deficit symptoms in this comorbid condition being related to the somatosensory gyrus rather than to action in the prefrontal cortex or reward system as in ADHD alone. Despite similar symptoms, the regions causing the symptoms, that is, the underlying brain morphology, may differ. It is thus possible that a diagnosis based solely on symptoms is not always in line with the relevant pathophysiology, and symptomatic diagnoses do not often lead to effective therapy. On the other hand, therapy based on pathogenesis, not on a symptomatic diagnosis, may lead to better outcomes. In cases where structural MRI shows abnormalities in the postcentral gyrus rather than the prefrontal cortex and the reward systems, as seen in this study, the effect of methylphenidate may be weaker. Such considerations may be helpful for the selection of medication in the context of prior therapeutic strategies.

The National Institute of Mental Health in the United States launched the Research Domain Criteria (RDoC) project as an answer to the problem that a diagnosis based on symptoms does not always lead to effective therapy. This project sought to create a framework for research on pathophysiology, especially in the fields of neuroscience and genomics^50^. Using this model, researchers and clinicians are expected to identify syndromes based on their pathophysiology and thus ultimately improve treatment outcomes for psychiatric diseases. Imaging studies may further help clarify the underlying neuronal mechanisms of psychiatric disorders and thus provide new frameworks for their classification. This may ultimately lead to the development of treatment strategies that consider underlying pathophysiology. Using this model, the field’s future understanding of conditions of the brain will undoubtedly improve, as will the precision of their treatment.

While our findings offer significant benefits to the field, our study has some limitations. First, it is limited by its inclusion of only patients with comorbid ASD and ADHD. To further investigate the more exact, detailed progress of these diseases, both combined and in isolation, additional longitudinal studies are required. Second, the present study does not include female patients with ASD + ADHD, and our findings can therefore not be generalized to all cases of ASD + ADHD. Third, some patients with ASD + ADHD in this study were under medication, for example with methylphenidate, which may have affected their brain structure^51^.

In the present study, we assessed the unique brain structure features of patients with ASD + ADHD. This approach, to the best of our knowledge, has not been used elsewhere, and the relatively large sample size used here presents a significant advantage. Patients with ASD + ADHD exhibited significantly decreased left postcentral gyrus volumes compared to TD controls; however, the left postcentral gyrus volume in patients with ASD + ADHD was only significantly smaller in children and preadolescents, not in adolescents. These findings suggest that the pathophysiology of ASD + ADHD may be primarily related to somatosensory deficits and delayed maturation of the left postcentral gyrus. Our results improve the field’s current understanding of the neurobiological mechanisms underlying comorbid ASD and ADHD and may lead to the development of novel treatment strategies that consider the relevant pathophysiology.

## Supplementary information

Supplementary Materials
