# Supplementary material for: Structural brain abnormalities in children and adolescents with comorbid autism spectrum disorder and attention-deficit/hyperactivity disorder
Source: Transl Psychiatry. 2019 Dec 9;9:332. doi: 10.1038/s41398-019-0679-z (PMC6901569; doi:10.1038/s41398-019-0679-z)
Supplement: Supplementary file 1 — Supplementary Materials [file 41398_2019_679_MOESM1_ESM.docx]

Supplementary Materials

Table S1. Total brain volumes

| Structure (mm^3^) | TD | | ASD+ADHD | | *p* (Uncorrected) |
| --- | --- | --- | --- | --- | --- |
|  | Mean | SD | Mean | SD |  |
| Total intracranial volume | 1497055.7 | 152646.2 | 1483807.7 | 151714.8 | 0.722 |
| Total grey matter volume | 778197.3 | 55752.5 | 764892.6 | 61100.3 | 0.521 |
| Cortex volume | 593851.1 | 47556.6 | 581445.4 | 52016.4 | 0.366 |
| Subcortical grey matter volume | 60532.2 | 4413.9 | 60388.8 | 4643.5 | 0.502 |
| Left-cerebellum-cortex volume | 61002.7 | 5721.5 | 60658.5 | 7242.8 | 0.904 |
| Right-cerebellum-cortex volume | 61017.8 | 5348.2 | 60664.3 | 6596.5 | 0.592 |

TD, typically developing; ASD, autism spectrum disorder; ADHD, attention-deficit/hyperactivity disorder; SD, standard deviation

**Table S2. Volumes of subcortical regions**

| Structure (mm^3^) | Lateralization | TD | | ASD+ADHD | | *p* (Uncorrected) | *p* (FDR-corrected) |
| --- | --- | --- | --- | --- | --- | --- | --- |
|  |  | Mean | SD | Mean | SD |  |  |
| Accumbens area | Left | 583.6 | 108.7 | 570.7 | 131.9 | 0.574 | 0.993 |
|  | Right | 606.9 | 101.1 | 607.2 | 109.3 | 0.659 | 0.993 |
| Amygdala | Left | 1640.1 | 209.9 | 1632.3 | 214.8 | 0.995 | 0.995 |
|  | Right | 1834.1 | 220.3 | 1800.8 | 205.4 | 0.261 | 0.993 |
| Caudate | Left | 3787.6 | 406.5 | 3767.2 | 504.3 | 0.871 | 0.995 |
|  | Right | 3845.7 | 414.2 | 3793.5 | 469.1 | 0.511 | 0.993 |
| Hippocampus | Left | 4193.7 | 352.6 | 4184.7 | 437.7 | 0.784 | 0.993 |
|  | Right | 4418.0 | 375.2 | 4376.0 | 431.5 | 0.771 | 0.993 |
| Pallidum | Left | 1999.2 | 264.0 | 1972.4 | 218.8 | 0.593 | 0.993 |
|  | Right | 1942.8 | 260.5 | 1943.9 | 253.0 | 0.466 | 0.993 |
| Putamen | Left | 5320.9 | 620.6 | 5334.1 | 692.2 | 0.477 | 0.993 |
|  | Right | 5392.4 | 672.7 | 5338.1 | 771.5 | 0.922 | 0.995 |
| Thalamus | Left | 7968.3 | 784.3 | 7954.5 | 765.9 | 0.691 | 0.993 |
|  | Right | 7522.1 | 727.9 | 7559.8 | 658.6 | 0.100 | 0.993 |
| Anterior corpus callosum |  | 810.0 | 114.8 | 821.1 | 124.6 | 0.346 | 0.993 |
| Middle anterior corpus callosum |  | 592.7 | 161.9 | 607.9 | 144.1 | 0.426 | 0.993 |
| Central corpus callosum |  | 627.9 | 154.2 | 647.0 | 142.2 | 0.145 | 0.993 |
| Middle posterior corpus callosum |  | 478.3 | 101.5 | 480.1 | 107.1 | 0.981 | 0.995 |
| Posterior corpus callosum |  | 882.7 | 149.9 | 874.1 | 141.3 | 0.477 | 0.993 |

TD, typically developing; ASD, autism spectrum disorder; ADHD, attention-deficit/hyperactivity disorder; SD, standard deviation; FDR, false discovery rate

**Table S3. Volumes of regions acquired using the Desikan-Killiany atlas template**

| Structure (mm^3^) | Lateralization | TD | | ASD+ADHD | | *p* (Uncorrected) | *p* (FDR-corrected) |
| --- | --- | --- | --- | --- | --- | --- | --- |
|  |  | Mean | SD | Mean | SD |  |  |
| Bankssts | Left | 3050.1 | 617.6 | 3007.2 | 576.0 | 0.9801 | 0.980 |
|  | Right | 2696.6 | 441.6 | 2623.7 | 450.0 | 0.3417 | 0.768 |
| Caudal anterior cingulate | Left | 2042.8 | 532.1 | 1942.1 | 519.0 | 0.2707 | 0.735 |
|  | Right | 2507.3 | 576.0 | 2438.3 | 623.6 | 0.4151 | 0.768 |
| Caudal middle frontal | Left | 7768.6 | 1378.1 | 7374.1 | 1384.6 | 0.0946 | 0.536 |
|  | Right | 7586.2 | 1453.0 | 7464.9 | 1593.7 | 0.7365 | 0.929 |
| Cuneus | Left | 3783.3 | 549.7 | 3729.7 | 630.8 | 0.4848 | 0.768 |
|  | Right | 4269.9 | 704.5 | 4238.7 | 692.6 | 0.8417 | 0.943 |
| Entorhinal | Left | 1895.7 | 358.7 | 1950.1 | 445.0 | 0.2930 | 0.738 |
|  | Right | 1847.0 | 353.5 | 1779.0 | 352.4 | 0.3703 | 0.768 |
| Frontal pole | Left | 1355.9 | 228.1 | 1362.5 | 222.6 | 0.8880 | 0.943 |
|  | Right | 1566.6 | 247.5 | 1563.5 | 269.4 | 0.7426 | 0.929 |
| Fusiform | Left | 11431.3 | 1382.1 | 11223.0 | 1663.7 | 0.3425 | 0.768 |
|  | Right | 11107.0 | 1491.1 | 10992.1 | 1670.9 | 0.5703 | 0.843 |
| Inferior parietal | Left | 16773.1 | 2561.9 | 16322.0 | 2602.2 | 0.3506 | 0.768 |
|  | Right | 19413.1 | 2699.0 | 18821.1 | 2932.2 | 0.2035 | 0.659 |
| Inferior temporal | Left | 13726.4 | 2200.2 | 13672.1 | 2320.7 | 0.6958 | 0.929 |
|  | Right | 12949.7 | 1906.7 | 12856.6 | 2231.7 | 0.9063 | 0.946 |
| Insula | Left | 8335.9 | 782.2 | 8306.3 | 943.8 | 0.4762 | 0.768 |
|  | Right | 8312.3 | 856.4 | 8249.1 | 876.6 | 0.5928 | 0.858 |
| Isthmus cingulate | Left | 3452.2 | 606.8 | 3440.9 | 511.2 | 0.9614 | 0.976 |
|  | Right | 3245.5 | 498.5 | 3251.1 | 473.0 | 0.7977 | 0.943 |
| Lateral occipital | Left | 15417.5 | 2061.2 | 15317.8 | 2185.4 | 0.8370 | 0.943 |
|  | Right | 15860.4 | 2083.9 | 15648.4 | 2228.1 | 0.9178 | 0.946 |
| Lateral orbitofrontal | Left | 9112.6 | 1170.5 | 8784.9 | 1154.7 | 0.0234* | 0.333 |
|  | Right | 8976.7 | 1161.5 | 8729.4 | 1116.9 | 0.0343* | 0.333 |
| Lingual | Left | 7805.3 | 1200.2 | 7941.9 | 1450.9 | 0.2809 | 0.735 |
|  | Right | 8379.2 | 1300.5 | 8388.8 | 1494.4 | 0.4968 | 0.768 |
| Medial orbitofrontal | Left | 6443.9 | 893.8 | 6219.4 | 795.6 | 0.0741 | 0.536 |
|  | Right | 6744.3 | 867.0 | 6527.3 | 745.1 | 0.1129 | 0.569 |
| Middle temporal | Left | 14261.8 | 2183.2 | 13832.6 | 2171.4 | 0.1533 | 0.579 |
|  | Right | 15287.9 | 1836.1 | 14875.4 | 2267.3 | 0.1341 | 0.570 |
| Paracentral | Left | 4477.0 | 517.8 | 4321.2 | 595.7 | 0.0307* | 0.333 |
|  | Right | 4905.5 | 642.8 | 4810.4 | 634.0 | 0.4777 | 0.768 |
| Parahippocampal | Left | 2145.1 | 303.0 | 2103.4 | 278.5 | 0.6757 | 0.929 |
|  | Right | 1959.3 | 302.1 | 1959.0 | 290.2 | 0.4813 | 0.768 |
| Pars opercularis | Left | 5917.8 | 1071.7 | 5859.5 | 1301.2 | 0.7630 | 0.929 |
|  | Right | 5085.0 | 967.4 | 4989.5 | 1085.6 | 0.7622 | 0.929 |
| Pars orbitalis | Left | 2823.4 | 553.1 | 2800.0 | 540.7 | 0.8545 | 0.943 |
|  | Right | 3325.8 | 524.7 | 3313.1 | 633.0 | 0.8877 | 0.943 |
| Pars triangularis | Left | 4933.9 | 786.1 | 4818.7 | 794.8 | 0.4014 | 0.768 |
|  | Right | 5880.0 | 1022.3 | 5558.5 | 1086.5 | 0.0299* | 0.333 |
| Pericalcarine | Left | 2224.4 | 469.5 | 2292.4 | 565.2 | 0.2552 | 0.723 |
|  | Right | 2565.3 | 557.1 | 2567.3 | 658.4 | 0.8763 | 0.943 |
| Postcentral | Left | 12373.0 | 1664.4 | 11517.2 | 1507.2 | 0.0003*** | 0.018* |
|  | Right | 11856.3 | 1563.6 | 11511.0 | 1691.5 | 0.1920 | 0.653 |
| Posterior cingulate | Left | 4012.8 | 541.9 | 3865.0 | 598.7 | 0.1254 | 0.569 |
|  | Right | 4148.8 | 625.4 | 4092.2 | 613.4 | 0.5522 | 0.834 |
| Precentral | Left | 16173.6 | 1565.5 | 15770.5 | 1928.7 | 0.1205 | 0.569 |
|  | Right | 15701.0 | 1751.8 | 15768.2 | 1722.3 | 0.4536 | 0.768 |
| Precuneus | Left | 13322.3 | 1705.4 | 12979.9 | 1426.4 | 0.4859 | 0.768 |
|  | Right | 13841.8 | 1650.2 | 13413.7 | 1617.4 | 0.1727 | 0.618 |
| Rostral anterior cingulate | Left | 3042.4 | 584.8 | 2916.8 | 645.0 | 0.1478 | 0.579 |
|  | Right | 2381.7 | 480.1 | 2227.2 | 507.6 | 0.0278* | 0.333 |
| Rostral middle frontal | Left | 20758.1 | 2945.0 | 20363.0 | 3627.5 | 0.4617 | 0.768 |
|  | Right | 21624.0 | 3477.9 | 20840.6 | 3533.9 | 0.4244 | 0.768 |
| Superior frontal | Left | 28695.1 | 2748.2 | 28066.8 | 3162.4 | 0.2301 | 0.680 |
|  | Right | 27126.2 | 3092.0 | 26971.1 | 3448.6 | 0.6595 | 0.929 |
| Superior parietal | Left | 17364.6 | 2618.3 | 16923.6 | 2320.8 | 0.4097 | 0.768 |
|  | Right | 17189.1 | 2180.8 | 16485.3 | 2222.2 | 0.0255* | 0.333 |
| Superior temporal | Left | 14770.0 | 1848.7 | 14758.5 | 2015.4 | 0.7148 | 0.929 |
|  | Right | 14036.2 | 1625.8 | 13945.7 | 1637.5 | 0.8164 | 0.943 |
| Supramarginal | Left | 15238.1 | 2416.1 | 14490.7 | 2499.3 | 0.0801 | 0.536 |
|  | Right | 13199.4 | 1978.5 | 12638.3 | 1957.0 | 0.0482* | 0.410 |
| Temporal pole | Left | 2391.7 | 557.3 | 2492.1 | 547.2 | 0.2268 | 0.680 |
|  | Right | 2422.8 | 560.9 | 2564.0 | 605.2 | 0.0928 | 0.536 |
| Transverse temporal | Left | 1384.2 | 223.0 | 1378.9 | 248.0 | 0.7650 | 0.929 |
|  | Right | 1106.3 | 164.4 | 1112.0 | 181.2 | 0.4617 | 0.768 |

Bankssts, banks of the superior temporal sulcus; TD, typically developing; ASD, autism spectrum disorder; ADHD, attention-deficit/hyperactivity disorder; SD, standard deviation; FDR, false discovery rate

**p* < 0.05; ****p* < 0.001

**Table S4. Cortical thickness of regions acquired using the Desikan-Killiany atlas template**

| Structure (mm) | Lateralization | TD | | ASD+ADHD | | *p* (Uncorrected) | *p* (FDR-corrected) |
| --- | --- | --- | --- | --- | --- | --- | --- |
|  |  | Mean | SD | Mean | SD |  |  |
| Bankssts | Left | 2.77 | 0.18 | 2.79 | 0.18 | 0.190 | 0.979 |
|  | Right | 2.90 | 0.19 | 2.85 | 0.19 | 0.027* | 0.750 |
| Caudalanteriorcingulate | Left | 2.97 | 0.25 | 2.98 | 0.23 | 0.982 | 0.993 |
|  | Right | 2.84 | 0.26 | 2.86 | 0.25 | 0.749 | 0.979 |
| Caudalmiddlefrontal | Left | 2.82 | 0.13 | 2.78 | 0.13 | 0.054 | 0.750 |
|  | Right | 2.82 | 0.12 | 2.81 | 0.15 | 0.868 | 0.979 |
| Cuneus | Left | 2.11 | 0.17 | 2.12 | 0.18 | 0.834 | 0.979 |
|  | Right | 2.11 | 0.16 | 2.14 | 0.16 | 0.147 | 0.979 |
| Entorhinal | Left | 3.17 | 0.34 | 3.21 | 0.32 | 0.816 | 0.979 |
|  | Right | 3.24 | 0.34 | 3.20 | 0.33 | 0.410 | 0.979 |
| Frontalpole | Left | 3.19 | 0.30 | 3.24 | 0.34 | 0.221 | 0.979 |
|  | Right | 3.15 | 0.31 | 3.10 | 0.34 | 0.526 | 0.979 |
| Fusiform | Left | 2.93 | 0.11 | 2.93 | 0.13 | 0.658 | 0.979 |
|  | Right | 2.92 | 0.11 | 2.90 | 0.13 | 0.160 | 0.979 |
| Inferiorparietal | Left | 2.73 | 0.15 | 2.73 | 0.16 | 0.757 | 0.979 |
|  | Right | 2.71 | 0.14 | 2.71 | 0.15 | 0.993 | 0.993 |
| Inferiortemporal | Left | 3.00 | 0.14 | 3.01 | 0.16 | 0.745 | 0.979 |
|  | Right | 2.96 | 0.14 | 2.98 | 0.16 | 0.668 | 0.979 |
| Insula | Left | 3.23 | 0.18 | 3.25 | 0.17 | 0.828 | 0.979 |
|  | Right | 3.20 | 0.17 | 3.19 | 0.16 | 0.491 | 0.979 |
| Isthmuscingulate | Left | 2.66 | 0.19 | 2.68 | 0.21 | 0.651 | 0.979 |
|  | Right | 2.62 | 0.18 | 2.65 | 0.18 | 0.388 | 0.979 |
| Lateraloccipital | Left | 2.27 | 0.13 | 2.27 | 0.14 | 0.722 | 0.979 |
|  | Right | 2.37 | 0.14 | 2.34 | 0.14 | 0.062 | 0.750 |
| Lateralorbitofrontal | Left | 2.95 | 0.15 | 2.95 | 0.15 | 0.697 | 0.979 |
|  | Right | 2.92 | 0.15 | 2.93 | 0.16 | 0.621 | 0.979 |
| Lingual | Left | 2.27 | 0.16 | 2.28 | 0.16 | 0.674 | 0.979 |
|  | Right | 2.27 | 0.19 | 2.29 | 0.19 | 0.075 | 0.750 |
| Medialorbitofrontal | Left | 2.89 | 0.19 | 2.90 | 0.20 | 0.824 | 0.979 |
|  | Right | 2.92 | 0.16 | 2.95 | 0.17 | 0.106 | 0.928 |
| Middletemporal | Left | 3.08 | 0.15 | 3.07 | 0.18 | 0.230 | 0.979 |
|  | Right | 3.09 | 0.15 | 3.08 | 0.16 | 0.299 | 0.979 |
| Paracentral | Left | 2.75 | 0.14 | 2.72 | 0.17 | 0.316 | 0.979 |
|  | Right | 2.78 | 0.14 | 2.77 | 0.15 | 0.456 | 0.979 |
| Parahippocampal | Left | 2.72 | 0.26 | 2.73 | 0.26 | 0.752 | 0.979 |
|  | Right | 2.70 | 0.22 | 2.69 | 0.24 | 0.832 | 0.979 |
| Parsopercularis | Left | 2.86 | 0.16 | 2.83 | 0.15 | 0.207 | 0.979 |
|  | Right | 2.86 | 0.14 | 2.86 | 0.16 | 0.936 | 0.979 |
| Parsorbitalis | Left | 2.90 | 0.27 | 2.91 | 0.26 | 0.775 | 0.979 |
|  | Right | 2.93 | 0.22 | 2.93 | 0.23 | 0.968 | 0.993 |
| Parstriangularis | Left | 2.78 | 0.18 | 2.77 | 0.19 | 0.839 | 0.979 |
|  | Right | 2.80 | 0.17 | 2.78 | 0.17 | 0.309 | 0.979 |
| Pericalcarine | Left | 1.71 | 0.17 | 1.73 | 0.18 | 0.300 | 0.979 |
|  | Right | 1.70 | 0.19 | 1.73 | 0.20 | 0.066 | 0.750 |
| Postcentral | Left | 2.31 | 0.12 | 2.30 | 0.15 | 0.402 | 0.979 |
|  | Right | 2.30 | 0.12 | 2.29 | 0.16 | 0.607 | 0.979 |
| Posteriorcingulate | Left | 2.85 | 0.15 | 2.85 | 0.16 | 0.925 | 0.979 |
|  | Right | 2.77 | 0.15 | 2.79 | 0.14 | 0.618 | 0.979 |
| Precentral | Left | 2.74 | 0.14 | 2.71 | 0.16 | 0.056 | 0.750 |
|  | Right | 2.71 | 0.13 | 2.69 | 0.15 | 0.172 | 0.979 |
| Precuneus | Left | 2.73 | 0.11 | 2.73 | 0.14 | 0.883 | 0.979 |
|  | Right | 2.74 | 0.12 | 2.75 | 0.13 | 0.437 | 0.979 |
| Rostralanteriorcingulate | Left | 3.12 | 0.24 | 3.12 | 0.24 | 0.937 | 0.979 |
|  | Right | 3.23 | 0.24 | 3.24 | 0.27 | 0.772 | 0.979 |
| Rostralmiddlefrontal | Left | 2.72 | 0.13 | 2.69 | 0.14 | 0.073 | 0.750 |
|  | Right | 2.71 | 0.12 | 2.71 | 0.12 | 0.651 | 0.979 |
| Superiorfrontal | Left | 3.09 | 0.12 | 3.07 | 0.15 | 0.260 | 0.979 |
|  | Right | 3.06 | 0.12 | 3.07 | 0.15 | 0.936 | 0.979 |
| Superiorparietal | Left | 2.43 | 0.12 | 2.44 | 0.12 | 0.742 | 0.979 |
|  | Right | 2.44 | 0.11 | 2.45 | 0.13 | 0.366 | 0.979 |
| Superiortemporal | Left | 2.95 | 0.13 | 2.95 | 0.16 | 0.725 | 0.979 |
|  | Right | 2.99 | 0.15 | 3.01 | 0.17 | 0.365 | 0.979 |
| Supramarginal | Left | 2.80 | 0.14 | 2.78 | 0.14 | 0.160 | 0.979 |
|  | Right | 2.79 | 0.14 | 2.78 | 0.14 | 0.509 | 0.979 |
| Temporalpole | Left | 3.40 | 0.37 | 3.46 | 0.36 | 0.552 | 0.979 |
|  | Right | 3.44 | 0.42 | 3.53 | 0.41 | 0.318 | 0.979 |
| Transversetemporal | Left | 2.61 | 0.18 | 2.58 | 0.25 | 0.350 | 0.979 |
|  | Right | 2.62 | 0.21 | 2.61 | 0.24 | 0.892 | 0.979 |

Bankssts, banks of the superior temporal sulcus; TD, typically developing; ASD, autism spectrum disorder; ADHD, attention-deficit/hyperactivity disorder; SD, standard deviation; FDR, false discovery rate

**p* < 0.05

**Table S5. Surface area of regions acquired using the Desikan-Killiany atlas template**

| Structure (mm^2^) | Lateralization | TD | | ASD+ADHD | | *p* (Uncorrected) | *p* (FDR-corrected) |
| --- | --- | --- | --- | --- | --- | --- | --- |
|  |  | Mean | SD | Mean | SD |  |  |
| Bankssts | Left | 1113.0 | 201.1 | 1093.1 | 188.7 | 0.839 | 0.951 |
|  | Right | 966.1 | 139.3 | 951.2 | 148.0 | 0.666 | 0.903 |
| Caudalanteriorcingulate | Left | 638.8 | 140.4 | 611.0 | 142.4 | 0.280 | 0.903 |
|  | Right | 765.7 | 157.8 | 748.5 | 170.9 | 0.511 | 0.903 |
| Caudalmiddlefrontal | Left | 2470.4 | 429.4 | 2394.4 | 442.0 | 0.485 | 0.903 |
|  | Right | 2390.1 | 454.5 | 2359.5 | 508.2 | 0.942 | 0.956 |
| Cuneus | Left | 1673.9 | 179.7 | 1642.0 | 229.3 | 0.552 | 0.903 |
|  | Right | 1844.3 | 236.0 | 1809.1 | 255.0 | 0.598 | 0.903 |
| Entorhinal | Left | 436.1 | 82.3 | 440.9 | 91.2 | 0.276 | 0.903 |
|  | Right | 402.3 | 67.2 | 389.6 | 68.5 | 0.529 | 0.903 |
| Frontalpole | Left | 270.8 | 31.5 | 269.6 | 31.1 | 0.878 | 0.951 |
|  | Right | 324.9 | 36.7 | 326.0 | 40.3 | 0.523 | 0.903 |
| Fusiform | Left | 3385.7 | 380.8 | 3316.8 | 456.2 | 0.381 | 0.903 |
|  | Right | 3302.5 | 411.2 | 3253.7 | 460.1 | 0.540 | 0.903 |
| Inferiorparietal | Left | 5409.1 | 780.1 | 5297.4 | 847.9 | 0.864 | 0.951 |
|  | Right | 6206.3 | 837.7 | 6008.2 | 962.6 | 0.326 | 0.903 |
| Inferiortemporal | Left | 3695.2 | 554.7 | 3664.0 | 554.9 | 0.677 | 0.903 |
|  | Right | 3552.6 | 505.8 | 3486.0 | 513.9 | 0.728 | 0.903 |
| Insula | Left | 2530.0 | 250.8 | 2517.1 | 285.7 | 0.738 | 0.903 |
|  | Right | 2544.9 | 264.6 | 2541.8 | 310.6 | 0.678 | 0.903 |
| Isthmuscingulate | Left | 1137.1 | 178.0 | 1132.2 | 151.5 | 0.967 | 0.967 |
|  | Right | 1031.0 | 139.7 | 1020.4 | 130.9 | 0.806 | 0.940 |
| Lateraloccipital | Left | 6096.5 | 686.8 | 6088.9 | 847.6 | 0.320 | 0.903 |
|  | Right | 5893.9 | 732.5 | 5907.4 | 841.2 | 0.205 | 0.897 |
| Lateralorbitofrontal | Left | 2681.5 | 361.9 | 2581.7 | 325.7 | 0.045* | 0.498 |
|  | Right | 2733.1 | 386.1 | 2648.9 | 360.1 | 0.030* | 0.420 |
| Lingual | Left | 3169.5 | 443.0 | 3221.9 | 547.2 | 0.347 | 0.903 |
|  | Right | 3386.5 | 517.1 | 3369.9 | 618.4 | 0.910 | 0.951 |
| Medialorbitofrontal | Left | 1897.0 | 287.2 | 1820.6 | 254.2 | 0.063 | 0.498 |
|  | Right | 1927.3 | 258.1 | 1848.6 | 226.5 | 0.028* | 0.420 |
| Middletemporal | Left | 3539.1 | 487.1 | 3483.6 | 499.9 | 0.734 | 0.903 |
|  | Right | 3897.8 | 453.6 | 3797.0 | 527.7 | 0.323 | 0.903 |
| Paracentral | Left | 1463.0 | 153.1 | 1434.0 | 191.3 | 0.183 | 0.882 |
|  | Right | 1601.2 | 196.2 | 1579.8 | 197.4 | 0.861 | 0.951 |
| Parahippocampal | Left | 687.7 | 90.0 | 673.3 | 82.3 | 0.476 | 0.903 |
|  | Right | 642.1 | 95.0 | 643.1 | 92.5 | 0.490 | 0.903 |
| Parsopercularis | Left | 1773.2 | 326.6 | 1777.0 | 383.9 | 0.907 | 0.951 |
|  | Right | 1561.3 | 271.7 | 1537.7 | 312.9 | 0.942 | 0.956 |
| Parsorbitalis | Left | 703.8 | 109.5 | 691.8 | 105.1 | 0.552 | 0.903 |
|  | Right | 867.8 | 122.0 | 853.5 | 134.8 | 0.470 | 0.903 |
| Parstriangularis | Left | 1473.4 | 226.2 | 1450.3 | 238.8 | 0.612 | 0.903 |
|  | Right | 1770.9 | 301.7 | 1688.9 | 314.0 | 0.103 | 0.721 |
| Pericalcarine | Left | 1455.5 | 260.4 | 1487.0 | 331.7 | 0.419 | 0.903 |
|  | Right | 1686.5 | 312.8 | 1659.5 | 390.1 | 0.468 | 0.903 |
| Postcentral | Left | 4668.6 | 627.0 | 4392.4 | 562.9 | 0.002** | 0.140 |
|  | Right | 4493.0 | 560.9 | 4421.8 | 613.7 | 0.616 | 0.903 |
| Posteriorcingulate | Left | 1287.8 | 166.4 | 1247.1 | 197.0 | 0.227 | 0.903 |
|  | Right | 1337.7 | 203.4 | 1310.1 | 175.5 | 0.407 | 0.903 |
| Precentral | Left | 5296.0 | 516.0 | 5224.0 | 610.8 | 0.752 | 0.903 |
|  | Right | 5230.5 | 583.0 | 5301.8 | 578.5 | 0.030* | 0.420 |
| Precuneus | Left | 4427.0 | 583.4 | 4322.6 | 510.5 | 0.679 | 0.903 |
|  | Right | 4655.8 | 577.3 | 4472.8 | 577.5 | 0.064 | 0.498 |
| Rostralanteriorcingulate | Left | 836.9 | 172.7 | 798.9 | 179.2 | 0.177 | 0.882 |
|  | Right | 616.4 | 118.7 | 581.5 | 126.2 | 0.050 | 0.498 |
| Rostralmiddlefrontal | Left | 6415.9 | 961.2 | 6368.9 | 1079.1 | 0.685 | 0.903 |
|  | Right | 6777.0 | 1201.0 | 6544.1 | 1235.0 | 0.721 | 0.903 |
| Superiorfrontal | Left | 7874.4 | 840.8 | 7757.0 | 995.0 | 0.761 | 0.903 |
|  | Right | 7566.5 | 994.9 | 7477.3 | 1077.8 | 0.724 | 0.903 |
| Superiorparietal | Left | 6284.6 | 883.2 | 6138.5 | 853.8 | 0.583 | 0.903 |
|  | Right | 6199.0 | 747.8 | 5922.4 | 848.4 | 0.016* | 0.420 |
| Superiortemporal | Left | 4241.0 | 464.4 | 4238.0 | 521.6 | 0.669 | 0.903 |
|  | Right | 3990.5 | 413.0 | 3936.6 | 419.2 | 0.892 | 0.951 |
| Supramarginal | Left | 4820.8 | 761.4 | 4637.0 | 844.8 | 0.384 | 0.903 |
|  | Right | 4197.8 | 608.9 | 4065.4 | 582.7 | 0.189 | 0.882 |
| Temporalpole | Left | 467.3 | 65.5 | 474.8 | 62.8 | 0.178 | 0.882 |
|  | Right | 461.7 | 67.3 | 472.1 | 74.2 | 0.159 | 0.882 |
| Transversetemporal | Left | 481.9 | 72.3 | 491.0 | 80.4 | 0.413 | 0.903 |
|  | Right | 374.0 | 48.0 | 374.2 | 57.6 | 0.634 | 0.903 |

Bankssts, banks of the superior temporal sulcus; TD, typically developing; ASD, autism spectrum disorder; ADHD, attention-deficit/hyperactivity disorder; SD, standard deviation; FDR, false discovery rate

**p* < 0.05; ***p* < 0.01
